# ‘A Great Beneficial Disease’: Colonial Medicine and Imperial Authority in J.G. Farrell’s *The Siege of Krishnapur*

**DOI:** 10.1007/s10912-014-9313-5

**Published:** 2014-11-15

**Authors:** Sam Goodman

**Affiliations:** Weymouth House, The Media School, Bournemouth University, BH12 5BB Fern Barrow, Poole, Dorset UK

**Keywords:** Colonial medicine, British Empire, 1970s fiction, Post-war

## Abstract

This article examines J. G. Farrell’s depictions of colonial medicine as a means of analysing the historical reception of the further past and argues that the end-of-Empire context of the 1970s in which Farrell was writing informed his reappraisal of Imperial authority with particular regard to the limits of medical knowledge and treatment. The article illustrates how in *The Siege of Krishnapur* (1973), Farrell repeatedly sought to challenge the authority of medical and colonial history by making direct use of period material in the construction of his fictional narrative; by using these sources with deliberate critical intent, Farrell directly engages with the received historical narrative of colonial India, that the British presence brought progress and development, particularly in matters relating to medicine and health. To support these assertions the paper examines how Farrell employed primary sources and period medical practices such as the nineteenth-century debate between miasma and waterborne Cholera transmission and the popularity of phrenology within his novels in order to cast doubt over and interrogate the British right to rule. Overall the paper will argue that Farrell’s critique of colonial medical practices, apparently based on science and reason, was shaped by the political context of the 1970s and used to question the wider moral position of Empire throughout his fiction.

James Gordon Farrell (1935–79) is often considered one of post-war Britain’s most underrated novelists. Despite a successful career that spanned the 1960s and 1970s, Farrell has often been overshadowed by more famous contemporaries such as Angela Carter and John Berger. Lacking a literary movement under which to classify him and his work after his untimely death, Farrell’s popular reputation went into decline; though critically lauded by writers and reviewers such as Margaret Drabble and Malcolm Dean, his novels largely remained the preserve of dedicated admirers. Further, Farrell’s arguably finest work was in a genre that was for many years considered deeply unfashionable, namely the historical novel. However, in 2010 Farrell’s novel *Troubles* (1970) was awarded the Lost Booker Prize, making him one of only four people to be awarded the prize on more than one occasion and rekindling popular interest in his work.^1^ The award was in many ways a fitting tribute to an author whose career, oeuvre and chosen genre were shaped by the Booker. In the first decade of the Booker Prize For Fiction, six winning entries were historical novels (Strongman 2002). One of these, Farrell’s *The Siege of Krishnapur* (1973), was part of a loosely structured series including *Troubles* and *The Singapore Grip* (1978) which has become known as his ‘Empire’ trilogy.^2^

The Empire Trilogy consists of novels based around three key episodes from the history of the British Empire, all of which share a characteristic emphasis on decline. Representative of the after-Empire context of the 1970s in which he was writing, Farrell portrays Empire at key points of historical conflict: Anglo-Protestant society in Ireland on the brink of the Irish Civil War of 1922; an East India Company station during the Mutiny of 1857; and the final days of British colonial rule in Singapore before the Japanese invasion and occupation in 1942.^3^ These fictionalised re-engagements with history and received Imperial mythology are designed not to be nostalgic or celebratory but are instead written with a clear sense of critical intent as Farrell seeks to engage with the state of contemporary Britain through focus on its imperial past.

A key element in Farrell’s reappraisal of the nation’s colonial past, and similarly important in understanding the novelist himself, is his decision to include recurrent motifs of illness throughout his work. Critic John McLeod notes that ‘illness and its consequences are prevailing issues’ throughout Farrell’s career as a writer and that in each of his seven novels ‘key characters often suffer from debilitating or fatal conditions’ (2007, 5). Similarly, in *Troubled Pleasures: The Fiction of J.G. Farrell* (1997), Ralph Crane and Jennifer Livett recognise that ‘sickness (has) occupied a central position in Farrell’s novels’, describing it as ‘omnipresent’ in the later *Empire Trilogy* (1997, 126). Despite this identification of medical matters arising in Farrell’s work, the implications of such remain largely unexplored and untheorised beyond their thematic function--a critical gap that this article seeks to correct.

Farrell himself died relatively young, drowned off the Irish coast at the age of forty-four. A contributory factor to his death was a lack of mobility in his upper body, a lasting effect of polio contracted whilst at Oxford as an undergraduate.^4^ Previously an outstanding collegiate rugby player, between 1956–57 Farrell spent a number of weeks in an iron lung leaving him with life-long poor health and first-hand knowledge of human frailty. McLeod views this sudden and shocking experience as that which shapes Farrell’s sense of the ‘essential infirmity of human life,’ both in terms of physical well-being and social standing (2007, 5). However, whilst the sudden and life-altering effects of his own experience no doubt influenced the content of his writing as McLeod states, it would be too straightforward an analysis to suggest that Farrell’s interest in medicine was solely driven by autobiographical motives. Rather, illness and its effects possess a dual-purpose in Farrell’s fiction, serving to illustrate how rapidly the circumstances and certainties of life can alter and as a means of engaging with the cultural authority of the British Empire.

In this article I will argue that in *The Siege of Krishnapur*, illness and medicine provide Farrell with an opportunity to engage with the dramatically altered circumstances of Britain and British authority particular to the 1970s. I will examine Farrell’s depictions of colonial medicine as a means of analysing the historical reception of the further past and argue that the end-of-Empire context in which Farrell was writing informed his reappraisal of Imperial authority with particular regard to the limits of medical knowledge and treatment. I will illustrate how Farrell repeatedly sought to challenge the authority of medical and colonial history by making direct use of period material in the construction of fictional narratives; by using these sources with deliberate critical intent, Farrell directly engages with the received historical narrative of colonial India, that the British presence brought progress and development, particularly in matters relating to medicine and health. Within each of his novels, Farrell posits a relationship between the assault on medical expertise and the concurrent attack on the British establishment in order to illustrate the limits and shortcomings of imperial principles. The article will be divided into three sections, beginning with an examination of the critical and cultural contexts that surround Farrell’s novel in order to illuminate why he chose to employ medicine not simply as a literary motif but as a means of active historical critique. The article will then consider the two major medical themes of the novel, cholera and phrenology, with reference to the ways in which Farrell uses them to explore and satirise ideas of British authority and expertise. Whilst Farrell’s engagement with issues of medicine and historiography spans the entire Empire Trilogy, for the purposes of this article I will confine my analysis to *The Siege of Krishnapur* though make reference to the other novels where relevant.

## Cultural and critical contexts

*The Siege of Krishnapur* takes place in a fictional East India Company station as it is suddenly caught up in the events of the Mutiny in the summer of 1857. The plot concerns the efforts of the British residents, particularly the Collector, Mr. Hopkins, an adherent of progress and industry obsessed with the Great Exhibition of 1851, and the Byronic poet George Fleury, to defend the garrison long enough for relief to arrive. Despite the relative simplicity of the novel’s plot, to consider Farrell an adherent of the realist imperial adventure novel in the style of his near contemporary John Masters would be to overlook the ironic and parodic intent of his work. Farrell’s objective in *The Siege of Krishnapur*, as he revealed in an interview shortly after the novel’s publication, was to create ‘a novel of ideas which could be read at the same time simply as an adventure story’ (Dean 1973, 11). Unlike a novelist such as Masters, Farrell’s work does not seek to present the values of Empire as a curative for the social ills of the 1970s. Instead, *The Siege of Krishnapur* is a novel about the continual clash of opposites: one of past and present, tradition and progress, the British Empire and India, and the Englishmen and Indians.

In order to explore both why and how Farrell would compose his novel in this way, it is important to preface analysis of *The Siege of Krishnapur* with some contextual information on the literary and cultural circumstances in which it was written. Though the significant historical and historiographical approaches of the post-war period are often linked to the so-called ‘cultural turn’ of the late 1960s, it was in the 1970s that the process of re-examining the modern and imperial past of Britain began in earnest. However, rather than this process of re-examination diminishing literary interest in history and the historical novel, the Booker Prize records are testament to the reverse; indeed, as A.S Byatt asserts in *On Histories and Stories: Selected Essays*, there was a ‘sudden flowering of the historical novel in Britain’ in the post-war period, representative of a wider authorial and public fascination with the rapidly fading world of Britain’s Imperial grandeur (2000, 9). Mariadele Boccardi argues that this trend is ‘a reaction against post-structuralist and postmodern arguments for the end of history’; however, given the destructive character that pervades Farrell’s work and recurrent use of illness as metaphor, his engagement with Britain’s Imperial past is wholly anti-nostalgic and one intent on re-examining history with as much scrutiny as either of these movements (2009, ii).

Similarly, Farrell’s decision to write a Mutiny novel twenty-six years after Indian independence means that it would be unwise, not to mention difficult, to separate entirely his work from the influence of contemporary scholarship of the 1970s. It has been argued by critics such as Peter Morey that Farrell is a postmodern author returning to British India to criticise colonialism through the blending of comedy and pathos (2000, 110). Given the politicised nature of Farrell’s undertaking as well as the inherent and inescapable racial context of Empire and British India, a range of postcolonial theories and analyses can be applied to *The Siege of Krishnapur* as well, in particular those that explore racial stratification and the classificatory techniques of British colonial society. However, such analyses must also be approached with caution so as not to erroneously and anachronistically label Farrell’s work wholly within postcolonial discourse.^5^ For whilst *The Siege of Krishnapur* is sensitive to the portrayal of the colonised subject, the novel draws its focus largely on the actions, thoughts and failings of the coloniser, that of the British garrison; again with emphasis on the temporal duality of Farrell’s novel, this focus is intended as a synchronic critique of empire and the popular conception of a key moment in imperial mythology, namely ‘the effect of colonization on the colonising power itself’ in both short and long term (Crane and Livett 1997, 99). Thus Farrell’s novel sits uneasily between paradigms; too parodic to be sincerely postcolonial and too sincere to be entirely postmodern. It should be recognised though that Farrell’s return, after Empire, to the days of colonial India in no way validates colonialism’s existence but rather exposes its limitations. This intent means that Farrell’s post-colonial return is not merely temporally and aesthetically after-Empire but engages in a critical pursuit of its legacy, quite literally ‘going after’ Empire through parodic and ironic critique.

In addition to postmodernism and postcolonialism, the other key theme of many historical novels of the 1970s was that of unrest--a direct response to the political turmoil of the decade.^6^ In his seminal *The Break-up of Britain Crisis and Neo-nationalism, 1965–75* (1979), Tom Nairn asserted that rapid decolonisation, economic ‘stagflation’ and the social and political conflict that characterised the 1970s were representative of a society undergoing a ‘slow-motion landslide’ of disintegration (62). Bart Moore-Gilbert’s analysis of 1970s fiction in which he argues that British fiction of the era gives ‘the impression of a society on the verge of social disintegration, or civil war’ seems particularly applicable to Farrell whose novels of the period all feature episodes of violent unrest and upheaval (1994, 152). Farrell’s Empire Trilogy repeatedly features episodes in which the British societies depicted are on the verge of moral and physical disintegration.

Examining the root causes of British decline is the driving force of Farrell’s narrative in *The Siege of Krishnapur*, and the novel illustrates a colonial culture preoccupied with notions of its own progress, primarily in medical, scientific and spiritual matters. Farrell’s choice of period, location and medium in which to conduct this examination is highly significant, as it represents the high-water mark of the British Imperial mission to India and its simultaneously civilising and industrialising intent (Harrison 2002, 154). Farrell’s critical approach thus becomes an attack on British naiveté as well as a cherished myth of Empire and nationhood. The irony of the British clinging to their noble principles and certainties of pre-eminence whilst under siege is exploited to great effect by Farrell throughout; indeed, some of the book’s most blackly comedic moments come as its characters extol Empire’s virtuous intent as their world quite literally falls to pieces around them amidst the sepoys’ cannonades. The language used to describe the perceived spread of Victorian enlightenment is commonly that of a medicalised, often physiological nature; as Fleury, who has travelled to India to research a tract on how Empire has hastened the development of native civilisation, states: ‘Besides Doctor, everyone I talk to in Calcutta about my book tells me to look at this or that…a canal that has been dug or a cruel practice like infanticide of suttee which has been stopped…and these are certainly improvements of course, but they are only symptoms, as it were of what should be a great beneficial disease’ (Farrell 2008, 38). Farrell’s use of medical idiom here is not just a literary motif but rather establishes medicine as a key component of the British presence in India, its authority bound up in scientific knowledge and its responsibility to improve the lives of its Indian subjects.

For a novel so clearly critical of Empire, Farrell’s own narrative voice is far more muted than one might imagine when it comes to the subject of British folly. Rather than choosing to ridicule openly his characters for their misguided beliefs with a direct authorial voice, Farrell allows the peculiarities of historical fiction to accomplish this for him. In order to give his satire its teeth, Farrell chooses to employ a range of eyewitness accounts and factual medical material as part of his unpicking of British history.^7^ For instance, Krishnapur, though an invention, is a portrait drawn from Farrell’s visits to Lucknow and Cawnpore in 1971 as detailed in his *India Diary*, which was posthumously published along with his unfinished novel *The Hill Station* in 1981. Similarly, the research that underpins Farrell’s historicised fiction is indicated in his ‘afterword’ in *The Siege of Krishnapur* in which he reveals his extensive use of diaries and letters from the Siege of Lucknow (June-September 1857), often repeated nearly verbatim in the text of the novel or whose authors serve as inspiration for many of his characters (McLeod 2007, 61). Chief among the accounts Farrell employs are those of the Reverend Henry S. Polehampton and Maria Germon, wife of an Army officer, both present during the Siege of Lucknow, and the testimony of Mark Thornhill, Company Collector at Muttra (Farrell 2007, 314).^8^ These figures, their testimonies and their experiences of privation, sickness and endurance recur throughout Farrell’s novel, not only providing the plot with dramatic set-pieces but also supplying a great deal of the grim comedy which pervades the narrative.

Much like his use of the narrative accounts of the Indian Mutiny, Farrell uses contemporary sources such as medical diaries, scholarly journals, scientific case-studies and newspaper articles from the 1850s as the basis for his text, reflecting their tone, style and structure throughout. Malcolm Dean relates that he found nineteenth-century copies of the *British Medical Journal* among Farrell’s papers, indicating he took pains to make his portrayal of disease as authentic and period-specific as possible (Farrell 1993, 197). Such research emphasises the relevance of Farrell’s novels to a changing medical climate. Farrell’s literary critique of Empire corresponds with the contextual emergence of the medical humanities; Ronald A. Carson has argued that the medical humanities are ‘a product of the turbulent ‘60s, when authority and expertise were being questioned and traditional ways of doing things were being challenged’ (2007, 322). Farrell, writing in the 1970s, conforms to this view with *The Siege of Krishnapur* in particular seeking to question the traditional narratives of the British presence in India and the perception of authority in British medical expertise. Further, in his unpublished diary Farrell records that he became ‘…interested in writing, largely I suppose as a sort of self-therapy’ (Farrell 1965, 69). Farrell’s novels are therefore not only relevant within their own context, both as a means of understanding how he came to terms with his own illness through literature and how the British nation sought to come to terms with the rapid decline of Empire by the same means, but are also relevant today as examples of how that process involved the intersection of medicine and literature on a textual and metatextual scale.

In ‘The Imprint of Recorded Events in the Narrative Form of J. G. Farrell’s The Siege of Krishnapur’ (1993), Lars Hartveit argues how the appeal of documents naturally intersected with Farrell’s literary intent and chosen genre. Indeed, in his own archival research, conducted after Malcolm Dean’s, Hartveit noted a file card marked ‘HEALTH’ among many others within Farrell’s papers at Trinity College Dublin, indicating his inclusion of medical material was in no way an incidental detail of his writing but rather a systematic and deliberate process of inclusion (1993, 458). Farrell’s reasoning behind such documentary faithfulness was his belief that history ‘leaves so much out…it leaves out the most important detail of all: what being alive is like’ (1993, 452) Hartveit’s analysis is based on the work of metahistorian Hayden White and, as such, he views Farrell’s decision to construct fiction from historical detail as a matter of ordering material in a different way, thereby equating the novelist and historian as two-sides of a narrative coin (1993, 452), further suggesting Farrell’s status as a multidisciplinary writer. However, Farrell’s editorial decisions as to what to include in his narrative are often in excess of the then traditional historian of Empire, and it is only on further inspection that the full extent of Farrell’s ‘borrowings’ from period material becomes clear.

So many of Farrell’s textual details and vignettes are repeated verbatim from his sources; from Polehampton, Farrell takes a ‘liver-coloured spaniel’, ‘a fallen woman in a dak bungalow’ and an argument between a Catholic and Church of England over a group of unidentifiable bodies and their burial rites (Polehampton 1858, 108–9; 113–115; 311). From Thornhill, he repeats the confusion over the sudden appearance of chapattis in the spring of 1857 (Thornhill 1884, 2). From Germon, he derives boils, apoplexy and the bidding of exorbitant sums on everyday items as food runs scarce (Germon 1870, 55; 83–84). Folding these details into *The Siege of Krishnapur*, as he does with similar primary sources in his other novels, illustrates Farrell’s feeling that conventional, documentary history is somehow inadequate or deficient in that its 'factual' demands means it lacks the emotional and physical elements literature is able to provide. Rather, the novel is more readily disposed to represent the effects and subjective experience of living through history or, as Farrell put it, ‘undergoing history,’ becoming more accessible as a result (McLeod 2007, 37). Recognising as subsequent critics have that the ‘British experience of India was intensely physical’ (Collingham 2001, 1), the chief means through which Farrell achieves this is through the embodiment of experience: the positioning of the body, its deficiencies and its treatment at the centre of his novels.

The effect of this process of inclusion is that through individual incidents, objects or identities, Farrell is able to criticise more than the artform or profession in the foreground; the technique enables him to critique the wider ideology and values of Empire in simultaneity. In Crane and Livett’s *Troubled Pleasures*, this process is labelled ‘interdiscursivity’ (1997, 87). Crane and Livett relate interdiscursivity to the novel’s penultimate chapter in which the relief force arrives at Krishnapur and the survivors are led away from the compound, comparing Farrell’s portrait with the real-life painting ‘The Relief of Lucknow’ (1859) by Thomas Jones Barker. However, the principles of interdiscursivity pervade many other scenes and chapters throughout the book, not especially those which feature medicine and the role of the doctor. As the following sections will illustrate, Farrell uses medical details as part of this process of interdiscursivity, moving his novels away from orthodox historical fiction and towards medical humanities; by employing medical treatment and practice in an Imperial adventure novel, Farrell not only criticises the historical record of Empire but attacks the foundation of science, medicine and reason on which the garrison of Krishnapur and Empire are based.

## Don’t drink the water – cholera

Among the expected instances of scurvy, malnutrition, amputation and nervous exhaustion that befall the garrison of Krishnapur, the two central subjects that form the bulk of Farrell’s medical discourse are that of cholera and phrenology. The inclusion of infectious diseases and pseudoscience within the novel are not, however, merely incidental plot points or means of inserting ‘instant’ authenticating detail; instead the inclusion of nineteenth-century debates over cholera transmission is integral to Farrell’s novel, a fact made clear in his personal papers and manuscripts housed in Trinity College, Dublin.^9^ In Farrell’s handwritten notes and drafts for *The Siege of Krishnapur,* he provides an early list of key scenes and plot points around which the novel’s narrative will pivot. Included alongside headings such as ‘The Last Great Battle Scene’ and ‘The Relief’ is the heading ‘The Great Cholera Controversy Scene,’ an indication of Farrell’s own assessment of how important the scene was within the novel’s structure and critical intent. Tensions and disagreements between characters regarding cholera simmer throughout the narrative as the contagion steadily spreads, intersecting with deliberately anachronistic debates over Darwinism, the potential for a moving daguerreotype and other innovations. However, unlike these sections of dialogue, which add humour and contribute to the novel’s warping of history and chronology, the ‘Great Cholera Debate’ is strictly factual and period-accurate.

Alongside the index card entitled ‘HEALTH’ noted by Hartveit, Farrell’s papers contain another entitled ‘CHOLERA’ and a range of photocopied, transcribed and typed material on medicine and health drawn from a variety of nineteenth-century sources. Farrell’s reading lists and notes are extensive and varied; indeed, both Dean and Hartveit make reference to Farrell’s research ethic when preparing a new manuscript, particularly how his research in the British Library and the Victoria and Albert Museum archive would take at least a year (Hartveit 1993, 451; Farrell 1993, 195). The inclusion of archival material is significant for a number of reasons. Firstly, the decision to ensure that his fictional portrayal of the Indian Mutiny was rooted in a factual basis indicates a desire for exactitude and authenticity on Farrell’s part that went beyond the traditions of his chosen form, the Imperial adventure story. This decision also complicates Farrell’s categorisation under either postcolonial or postmodern genres, as his use of this material in his novels neither fits wholly within Linda Hutcheon’s definition of historiographic metafiction (1988, 14), nor does it explicitly privilege subaltern voices (Spivak 1989). Secondly, the conflation of fictional and factual modes of writing, especially in his use of case notes from medical procedures and the language and idiom of nineteenth-century medical practice, means that Farrell’s novel represents an interface between medicine and literature and situates him within the discourse of medical humanities. The focus on cholera within *The Siege of Krishnapur* is a means through which Farrell is able to consider resistance to change in both a contemporary and historical setting specifically through a medical lens.

Farrell uses the conflict over cholera to illustrate a range of internal divisions and disagreements amongst the novel’s British characters. As the siege continues, the general rivalry between the garrison’s civil surgeon Dr. Dunstaple and the regimental surgeon Dr. McNab becomes more greatly pronounced, coming to a head over how best to treat cholera. When the Collector takes a tour of the sick bay, Dunstaple becomes enraged over McNab’s methods and demonstrates what he believes to be the proper form of treatment:

Suddenly, he seized the Collector’s wrist and dragged him across the ward to a mattress on which, pale as milk beneath a cloud of flies, a gaunt man lay shivering, stark naked. ‘How d’you think I cured this man? How d’you think I saved his life? The Collector offered no suggestions so the Doctor explained that he had used the best treatment known to medical science…the treatment which, for want of a specific, every physician worthy of the name accorded his cholera patients…calomel, opium and poultices. (165–166)

Farrell’s text echoes almost precisely reports from two sources: firstly, the *Medical Times & Gazette* of 1854, mentioned explicitly in his handwritten notes, and secondly, a report entitled ‘General Board of Health Report On the Different Methods of Treatment Pursued in Epidemic Cholera in the Provinces Throughout England and Scotland in 1854’ published in 1855 by the Royal College of Physicians. The first of these sources contains a number of articles and reports which detail contemporary debates, treatments and publications on cholera, including case histories of outbreaks in 1832, in London in 1849 and in Newcastle and London again in 1853, all of which are reflected in Farrell’s novel. More precisely, much of Dunstaple’s approach to treating cholera is drawn from an article entitled ‘Statistics of the cases of the Cholera Epidemic 1853’ by J. S. Pearse and Jeffrey A. Marston. Pearse and Marston assert that the immediate treatment of cholera should involve ‘…a warm bath, a blister to the spine and calomel’ (*Medical Times and Gazette* 1854, 130); Dunstaple, when arguing with McNab, states that ‘…we must consider means of counter-irritating the disease…Hence a warm bath perhaps, and a blister to the spine’ (Farrell 2007, 254). Through his repetition of and adherence to such methods of treatment, Farrell aligns Dunstaple with the official, established views of his age. Dunstaple further derides McNab as a ‘quack,’ his investigation of native medicine such as the use of burtunga ants to close wounds acting as evidence of his inability to practice (94).

Farrell’s exactitude in Dunstaple’s description of acceptable treatment for cholera suggests that ‘…On the Different Methods of Treatment Pursued in Epidemic Cholera’ along with other contemporary publications of a similar nature may well have also comprised part of his original research.^10^ ‘…On the Different Methods of Treatment Pursued in Epidemic Cholera’ written by Drs. Paris, Alderson, Babington, Tweedie and Bagshaw-Ward of the General Board of Health and part of the Treatment Committee of the Medical Council outlines four main treatments for cholera infection: alterative, astringent, stimulant and cathartic, concluding that ‘calomel and opium stand highest in success’ when used as part of the astringent method (Paris et al. 1855). Dunstaple again echoes almost exactly the report’s assertion that ‘cholera attacks the mucous lining of the intestinal canal’ but instead of using calomel and opium in the astringent method, he instead combines elements of alterative and stimulant forms, using chloroform and turpentine along with mustard-coated flannels. Incidentally, the Medical Board’s report concludes that the alterative and stimulant forms of treatment to have a 36.2% and 54% rate of death respectively. To put these statistics into perspective, they are nearly double and triple the death rate of the recommended treatment. Not for the first time, Farrell illustrates a gulf between the good intentions and the ill-effects of imperial methods.

Farrell later develops the dispute between Dunstaple and McNab from one of the initial disagreements over appropriate treatment into an open conflict over the miasma and water-borne theories of cholera. In the latter of two scenes, appropriately set in the garrison’s church as a means of emphasising and undermining the notions of faith and belief in both science and the British Christian mission, Dunstaple demands that McNab justify his hydration-based treatment of cholera patients. In this exchange, Dunstaple repeats his assertion that impure air is the only cause of cholera infection: ‘Dr Baly finds *the only theory satisfactorily supported by evidence* is that “which regards the cause of cholera as a matter of some process, whether chemical or organic, in impure or damp air”…Dr Dunstaple paused triumphantly for a moment to allow the significance of this to seep in’ (252; emphasis in original). Farrell again uses period sources, basing Dunstaple’s arguments on the ‘Report on Epidemic Cholera’ by Drs. William Baly and William Gull, Royal College of Physicians (1854), referenced in both the *Medical Gazette* of 1854 and the report by Drs. Paris et al., and McNab’s ripostes on the research undertaken by John Snow in London between 1853–54.

Despite employing the weight of Snow’s statistical analysis of the 1853 cholera epidemic, McNab’s counter-argument fails to convince the garrison of Krishnapur: ‘with the best will in the world and in ideal circumstances it is next to impossible to escape cerebral indigestion as someone quotes comparative figures as fluently as Dr McNab’ (266). The statistically-sound analyses and alternative forms of treatment conducted by Snow in London are shown to gain little traction in an Indian context; in this sense, Farrell’s novel is reflective of the ‘fiercely divided’ state of the Indian Medical Service (IMS), which continued to support miasma theory well into the early twentieth century, arguing that environmental pollution, bad air, poor sanitation as well as impure water were the source of cholera in India (Klein 1980, 30). Mark Harrison argues that Snow’s analysis asked only a limited range of questions and excluded a number of factors pertinent to India and colonial space (2002, 191). Indeed, key figures within the IMS, such as Dr. James Bryden, maintained that there were also distinct atmospheric and meteorological differences between Britain and India which affected the transmission and treatment of cholera (1869, 14). E. M. Collingham notes that European ideas and their proponents were not often afforded credibility by an IMS dominated by an older generation of doctors with out-moded concepts of the body and its treatment (2001, 90–91). For instance, in the 1870s, the Indian Sanitary Commissioner J. M. Cunningham condemned Snow’s ideas as ‘mere hypothesis’ based on ‘inexact and imperfect evidence’ (Klein 1980, 31); in *Krishnapur*, Dunstaple similarly derides McNab’s convictions as ‘rubbish’ and charges McNab as unable to provide proof of his theories beyond these statistics. Denying the analyses of McNab and by extension Snow, the garrison instead take comfort from the authority associated with Empire, again drawing comparison to Farrell’s contemporary moment in which he suggests that the British public are likewise denying the evidence before them and are instead looking backwards to the perceived securities of the past.

The point of Farrell’s faithful use of his source material here is explored in John Spurling’s essay entitled ‘As Does the Bishop.’ Spurling calls attention to the way in which Farrell’s choice of medium, the historical novel, allows him to ‘observe human nature more coolly and clearly, from a seat in the gods,’ more often than not through the use of dramatic irony (1993, 155). The immediate joke here is that for all Dunstaple’s passionate conviction and denunciation of the water-borne theory, the reader knows that miasmatic transmission of cholera is eventually disproven and that history will vindicate McNab. However, historical irony is not Farrell’s sole intention. The use of contemporary medical journals as the basis for Dunstaple and McNab’s dialogue again reveals the layering and nuance present in the author’s use of reciprocity and exchange particular to his historical novels, both in terms of literary form and theme. Instead of simply employing it as a plot point to illustrate tension, Farrell uses the cholera debate as a lens through which to satirise further the adherents of Empire in both Victorian society within the novel and contemporary society outside of it. As critics have observed, ‘when Farrell plays off the present against the past he is critical of both’ (Goonetilleke 2003, 412). Here, Farrell’s split perspective on cholera seeks not only to highlight the divisions inherent to empire both past and present, but also to draw a parallel with contemporary debate on how best to treat the metaphorical ills of the nation either through reliance on traditional, if not entirely successful methods, or by disregarding the past and embracing something new and unproven.

Farrell takes this reliance on tradition he views as endemic to the British character to ever more ridiculous extremes in order to illustrate both its redundancy in a modern context and the hollowness of its prestige. For example, when Dunstaple again explains the proper methods by which cholera should be treated he does so with revealing grandiloquence: ‘[t]o relieve the pains in the head we might order leeches to the temples. An accepted method of counter-irritation in cholera is with sinapisms applied to the epigastrium…or, if I must interpret these learned expressions for the benefit of my distinguished colleague, with mustard plasters to the pit of the stomach’ (254). Though the application of leeches and poultices appear archaic, Dunstaple’s ‘treatments’ were widely adhered to in contemporary colonial medicine; in a moment of relief in Maria Germon’s journal of her experiences at Lucknow, she notes with joy that her husband Charlie ‘brought over 6 bottles of mustard as we had very little and it was in great demand in cases of cholera’ (1870, 56).^11^ Moreover, the combination of leeches and poultices illustrate the central paradox of Farrell’s novel and the Victorian age he criticises, namely that the actions of its inhabitants illustrate faith in a world governed by logic and reason while exercising neither themselves; their enlightened principles are lost in a desire to retain their position. Farrell intimates that a preference for tradition against all better judgement is equally applicable in a 1970s context, as he stated in his interview with Dean: ‘I hoped to say something…about how we, in our thriving modern world of the 1970s hold our own ideas’ (Dean 1973, 12). By making his colonial Victorians ridiculous, Farrell strives not just to lampoon them and their views but also to make his contemporary audience consider how their own actions and judgments may one day too be assessed.

The unsettling effect of the oscillation between tradition and an uncertain alternative is keenly illustrated by Farrell. A source of much comedy during the debate over the treatment of cholera is the fact that the garrison ‘took to carrying cards in their pockets which gave the relevant instructions in case they should find themselves too far gone to claim the doctor they wanted’ (249). As both doctors hold forth on their respective theories, various members of the garrison cross-out and rewrite their preferred choice of doctor multiple times (representative of the constant swinging back and forth in opinion on medical treatment). Farrell captures a picture of Britons, to paraphrase Homi Bhabha, caught uncertainly in the act of composing themselves, indecisively caught between their perception of comfort and familiarity in tradition and the hard logic of reason and fact (2005, 70).

Farrell expands his metaphor to the doctors themselves, describing Dunstaple as ‘a kindly and paternal man’ possessing ‘authority and good humour…the more experienced, and hence more reliable of the two’ (250). McNab on the other hand, whilst similarly authoritative, ‘seldom smiled’ and ‘seemed to take a pessimistic view of your complaint, whatever it was’ (250). Farrell suggests that, quite understandably, the familiar, paternalistic view of Empire is preferable to the new, disquieting prospect of a post-imperial Britain, allowing one of his characters, the Magistrate, to observe with knowing glee ‘(H)ow much more easily they were swayed by prestige than by arguments!’ (253). However, Farrell further intimates that to continue to accept the rose-tinted, traditional view of Empire is a dangerous as belief in the effectiveness of mustard poultices as a cure for bacterial infection. As if to finally underscore the destructive potential of such passionate commitment to the past, Farrell has Dunstaple swallow a bottle of rice-water discharge gathered from a cholera patient in a bid to disprove McNab once and for all. In another example of deliberate anachronism akin to his mention of moving daguerreotypes, the episode in which Dunstaple deliberately drinks rice-water discharge is in reference to the near identical actions of Dr. Max Von Pettenkofer in 1892. An outspoken critic of Robert Koch and an opponent of bacteriology, Von Pettenkofer’s consumption of cholera bacilli proved an equally misguided act of defiance in the face of all evidence to the contrary (Klein, 1980, 45). However, Von Pettenkofer’s and Dunstaple’s actions nonetheless serve as illustrative examples of the power of conviction and the longevity of tradition in times of rapid change.

## Bringing matters to a head – phrenology

Alongside the mounting tensions over the treatment of cholera, another medical thread running through the novel is the veracity or otherwise of phrenology. Developed, as Farrell’s characters tell us, by Dr. Franz Joseph Gall of Vienna, phrenology was fashionable for the early part of the nineteenth century, particularly between the 1820s and 1840s (13). Although described as ‘a pseudo-science’ by reputable medical journals in the 1840s, the fashion for phrenology and belief in its principles remained popular throughout the nineteenth century and, in some instances, into the early twentieth (Hanen et al. 1980, 150). Phrenology, like cholera, becomes a similarly vital means by which Farrell is able to illustrate the manner in which outdated medical ideas were able to retain a degree of professional cachet in a colonial setting long after their abandonment in European medical discourses. The inclusion of phrenology enables Farrell not only to satirise British convictions in constant scientific and medical progress but also the actions and opinions of those who propagate such beliefs without recognition of their redundancy. Also, and perhaps more importantly, phrenology becomes one of the few instances in which Farrell’s depicts Indian characters in any detail other than as thinly drawn antagonists. As mentioned above, *The Siege of Krishnapur*, focuses mainly on the coloniser; the discussion that surrounds phrenological practice within the novel becomes a means of providing native characters with a more prevalent fictive voice, though, crucially, not in a language or context of their own. Phrenology is another European import to colonial space and more readily associated with control as part of the classificatory discourse of the coloniser; according to Lawrence James, phrenology was used in India to catalogue ‘criminal tribes’ (James 2007, 202).

Upholding this association with authority, phrenology’s greatest proponent among the garrison in *The Siege of Krishnapur* is Mr. Willoughby, the Magistrate. The novel contains repeated mentions of the Magistrate’s devotion to phrenology, whilst Farrell’s original manuscripts also include numerous extended sections or deletions from the finished text. Though apart from the Magistrate phrenology is largely discounted by other residents of Krishnapur, the fact that the subject remained popular amongst colonial practitioners of the period is clear, as the journal *Leaves Turned Down or the Autobiography of an Indian Army Surgeon* (1854) reveals. In this account, the anonymous army surgeon author confesses himself to be a devotee of phrenology and decides to conduct ‘an investigation on scientific principles’ on a Hindu Thugee prisoner reputed to have killed approximately 1,100 men over a period of fifty years (1854, 126). The Magistrate experiences a similar desire to get to grips with the skulls of his compatriots and of Indians alike; for instance, during a discussion about evacuating the garrison ‘the Magistrate was surveying the behaviour of the cantonment in an objective and utterly scientific manner…would there be a common skull-form that rejected it? He hungrily eyed heads, even neglecting his work to haunt the balder members of the cantonment’ (Farrell 1972, 102–103).

Farrell uses phrenology to satirise a number of Victorian concerns, particularly those related to the expression of female sexuality and the perceived effect of colonial space on morality (Ghosh 2006, 50). For example, the Magistrate fixates his attentions on the so-called ‘fallen woman’ of the garrison, Miss Lucy Hughes, recently jilted after an affair with an army officer.^12^ Convinced that Lucy’s ‘organ of Amativeness was extraordinarily well developed,’ namely the part of the brain alleged to control sexual desire, Farrell describes how the Magistrate had ‘been in the position of a scientist who has made a discovery which he knows to be true but is unable to prove’ (241). Amativeness appears to have been integral to Farrell’s decision to include phrenology in his novel; of the five index cards on phrenology amongst his papers, one is devoted entirely to notes and descriptions of amativeness. Chancing upon Lucy alone, the Magistrate finally makes his move just after the siege is relieved. In a reversal of a traditional romantic denouement expected of an Imperial adventure novel:

The Magistrate put a companionable hand on her shoulder and then, after a moment’s hesitation, slipped it onto the back of her neck. Perhaps Lucy would have melted weakly into his bony arms had not an expression of dismay and incredulity come over his face. She promptly slapped him as hard as she could, which was not very hard. She did not know what the matter was but knew instinctively that this was the right thing to do…the Magistrate, mortified, had made himself scarce. (310)

Beyond the comedy to be found in the play on Lucy’s natural and social instincts, the Magistrate’s shock at finding his principles challenged echoes the situation experienced by the surgeon in *Leaves Turned Down*. During his exchange with the Thugee prisoner, he finds the old man to possess the ‘best of bumps’; this throws him into some turmoil, leading him to doubt his phrenological devotion: ‘that night, phrenology and I had a strong tussle; and she had much difficulty in re-establishing herself in my estimation for this specimen of ‘mild Hindoo’ had given her the lie direct’ (1854, 126–128). Farrell uses phrenology as a means of exposing the hollowness of British convictions. In direct transposition of the wider conflict of the novel, Farrell equates the Magistrate’s ardent, if erroneous, belief in phrenology with belief in the benefits of British rule to India. The Magistrate’s discovery and flight indicates, at least initially, either a realisation of the shortcomings of phrenology and with it the British claim on medical and civilising authority or even a sense of horror at what his investigation reveals.

However, Farrell’s novel is ambiguous and does not offer conclusive proof that the Magistrate is forced to re-evaluate his beliefs. Again, much like in *Leaves Turned Down* where the anonymous author convinces himself that phrenology was not at fault after all, Farrell’s manuscript fragments suggests the same possibility with regards to the Magistrate: ‘[T]he Magistrate was aware that it was not phrenology that was in error, but his own inadequate use of it’ (Farrell 1972, 70). Though the Magistrate is surprised by his discovery, there is no concrete suggestion that he will re-evaluate his convictions. Of course, the British presence in India continued long after the Mutiny was put down, dissolving the East India Company and instead reinforced as a Crown endeavour. Farrell illustrates the resilience of long-held beliefs and the longevity of their consequences, even after compelling evidence to disprove them. In his decision to delete this section from the published version, however, Farrell makes the ambiguity inherent to the ending of *The Siege of Krishnapur* potentially hopeful, suggesting that even the strongest beliefs are subject to change if given the necessary impetus.

Farrell’s ambiguity runs deeper when considered in relation to Hari, the Maharajah’s son imprisoned by the Collector throughout the siege and one of few native characters seen up close or given dialogue.^13^ Imprisoned allegedly to ensure his safety as well as for his value as a hostage, Farrell characterises Hari as susceptible to British instruction; he is first presented eating a boiled egg whilst reading *Blackwood’s Magazine* (71) and represents the class of Anglicised Indians encouraged by Thomas Babington Macaulay’s *Minute on Education in India*: ‘a class of persons, Indian in blood and colour, but English in taste, in opinions, in morals, and in intellect’ (Jayapalan 2005, 56). Hari’s characterisation is significant for its evocation of mimicry and embodied racial difference. The chapter in which Fleury meets Hari and is given a tour of the palace is focalised through Fleury, allowing Farrell to present and critique the latent racism of the supposedly enlightened coloniser. Fleury notes with disconcertion that both Hari and the paintings of the previous Maharajahs that adorn the palace walls are ‘really…just one face…repeated again with varying skill’ (71). Fleury here repeats and rearticulates the prejudices of the British garrison, revealing both the tendency to homogenise the figure of the Indian and also the extent to which mimicry acts as an unsettling force upon the coloniser. As Bhabha argues in *The Location of Culture*, an image, and by extension here Hari himself, ‘can neither be ‘original’ – by virtue of the act of repetition that constructs it – nor ‘identical’ – by virtue of the difference that defines it’ (2004, 106). Hari is thus left paradoxically with and without form; his identity another site of contestation between British and Indian control.

Farrell’s inclusion of a character such as Hari affirms Morey’s suggestion that the relationship between coloniser and colonised is configured as a site of unequal exchange (2000, 11). Hari’s imprisonment ensures that the Maharajah’s forces do not attack; whilst the British improve their own position, it is at the cost of Hari’s liberty. Farrell takes the idea of unequal exchange further, however, through Hari’s interactions with the Magistrate. During his captivity, Hari is given a book on phrenology by the Magistrate and becomes a devotee of what he calls ‘Frenloudji…science of head,’ practicing his new found discipline on his Prime Minister, who has been taken into captivity with him (177). Hari too is absorbed by the study of amativeness, stating that ‘amativeness…the faculty gives rise to sexual feeling…not very powerful organ in Prime Minister. In me, very powerful. In Father it is fearfully, fearfully powerful so that all other organs wither away’ (177–178). Farrell illustrates how phrenology is used to classify and construct the figure of the native; Hari confirms and labels himself under the stereotype of the sexually licentious oriental (Said 2003, 4). The irony inherent to Farrell’s portrayal of Hari is that in a novel whose characters are convinced of the good they are bringing to India, the only thing that the native receives in the course of his contact with the British is his devotion to ‘Frenloudji;’ the much lauded, so-called civilising principles of Empire instruct Hari in a falsehood and serve only to make him complicit in his own denigration (177).

Beneath the surface comedy of Hari’s Pidgin English, itself double-edged, Farrell’s inclusion of phrenology in his novel represents a powerful indictment of the British Empire in India and the contemporary legacy of Imperial power. The component parts of phrenology, ‘phren’ from the Greek for mind and ‘logos’ as language, represent two elements of both history and contemporary modernity with which Farrell seeks to engage. Indeed, Farrell invites the reader to investigate the minds of his characters as they investigate those of others; phrenology becomes another of Farrell’s means of examining and satirising the mind of the Imperial Briton and the way that mind expresses not only its hopes, desires and fears but also its perception of itself and the world around it through fiction. Moreover, by highlighting the shortcomings of Victorian and Imperial values, Farrell seeks to counter any nostalgia for Empire in the post-Imperial 1970s. By making such an obvious example of pseudoscience a dominant theme in the novel, Farrell’s subversion of Victorian scientific and medical progress and the civilising principles of Empire is clear; for Farrell, the only lasting effects that the ‘great beneficial disease’ of British imperialism leaves on the Indian body are bullets, subjugation and quackery.

## Conclusions

Though his choice of medical matters is varied, the disputes over Phrenology and Cholera are united by Farrell’s central narrative intention, namely to strike at the heart of what Umberto Eco identifies as *Endoxa*, the accepted understanding of values and morals as propagated by dominant culture (1979, 161). By interlinking postmodern and postcolonial concerns within the format of the historical novel, Farrell is able to engage parodically with the history of British colonial India as a means of exploring the contemporary crisis of Englishness provoked by the failure of Empire after the Second World War (Crane and Livett 1997, 99). Rather than the received historical narrative of British unity in the face of external assault, in this instance the mutinous sepoys, Farrell recasts the Krishnapur garrison as inherently divided. By doing so, he posits that the disintegration of Empire is not a uniquely contemporary phenomenon but an historical one. The dramatic irony Farrell uses at a surface level gives way to a more tragicomic mode of transmission, one in which the reader is aware that the efforts of the garrison, indeed the efforts of Empire, will one day count for little. It is telling that during one chapter when the Collector turns his mind to what Empire may be like a hundred years hence in 1957, Farrell describes his usually formidable mental powers as suddenly enfeebled.^14^

By weaving factual source material into the fictional fabric of his novel, Farrell is using the historical novel to play complex games with time and place, position and perspective. The enjoyment for Farrell’s readership is in their knowledge that Phrenology, along with the airborne theory of Cholera transmission, is debunked in time; however, his characters remain largely convinced of their validity. By using medicine in this way, Farrell is able to turn the satire back to face his contemporary audience, and by mocking something such as the misguided adherence to the pseudo-science of phrenology, Farrell is inviting his readers to examine more closely what contemporary history in the 1970s was doing to the record of British Imperialism and its potential effect on their own adherences or beliefs. Moreover, it is significant that Farrell’s novel offers no resolution in the dispute over either cholera or phrenology; Dr. Dunstaple dies inconclusively of a heart attack whilst the Magistrate and Hari are not seen again after their respective exits from the narrative. Again, he draws a parallel between the past and the 1970s present in that there are no certainties and that no definitive endings will be forthcoming. Farrell suggests that a narrative with such history still has time to run.

## Endnotes

- ^1^ The other three authors being J.M. Coetzee, Peter Carey and, latterly, Hilary Mantel.
- ^2 ^Farrell had begun work on a fourth novel by the time of his death, posthumously published as *The Hill Station* in 1981. Set in India in 1871 and featuring many of the characters from *The Siege of Krishnapur*, it effectively makes Empire trilogy into a series. However, because of its incomplete nature, it is not always considered canonical by Farrell scholars.
- ^3 ^The term ‘Mutiny’ is used throughout this piece in reflection of its contemporary lexical currency at Farrell’s time of writing and not in any opposition to its modern one. The Mutiny of 1857 is now largely referred to as ‘the Rebellion of 1857’ or the ‘First War of Independence’.
- ^4 ^See Lavinia Greacen’s *J. G. Farrell: The Making of a Writer* (London: Bloomsbury, 2000) for further detail on Farrell’s early life.
- ^5 ^Francis B. Singh’s article, ‘Progress and History in J. G. Farrell’s *The Siege of Krishnapur*’ (*Chandrabhaga* 2 1979, 23–29) notes the generally poor presentation of native subjects in the novel and as such does not categorise Farrell as postcolonial.
- ^6 ^Farrell’s first instalment of Empire Trilogy is of course called *Troubles*; a title with a particular relevance and resonance in the 1970s. Intending to continue this trend, *The Siege of Krishnapur* was originally entitled ‘Difficulties’; See A. Johnson, ‘Ghosts of Irish Famine in J. G. Farrell's *The Siege of Krishnapur*’ *The Journal of Commonwealth Literature*, Vol. 46, (New York: Sage, 2011), pp. 275–292.
- ^7 ^In his 1973 interview with Malcolm Dean, Farrell expressed how he ‘had thought of constructing the novel entirely from contemporary nineteenth-century insights and observations’; Dean, ‘An Insight Job,’ p. 11.
- ^8 ^Germon and her husband both survived the siege of Lucknow. Polehampton’s journal states that he succumbed to ‘fever’ in July of 1857, though it is strongly implied by the editors of his diaries that it was most likely cholera that killed him, a fact later corroborated by G. W. Forrest’s *A History of the Indian Mutiny – Reviewed and Illustrated from Original Documents*, Vol. I (London: William Blackwood & Sons, 1904).
- ^9 ^I would like to acknowledge that my own inspection of Farrell’s papers was made possible through generous support from a Wellcome Trust Small Grant (grant number: 100559/Z/12/Z) awarded in October 2012 and the kind permission of Trinity College Library, Dublin.
- ^10 ^Dr. Dunstaple later mentions the report by ‘Dr. Baly’ directly; it is also referenced in the article by Paris *et aI* and reproduced in the *Medical Gazette* of 1854 referred to in Farrell’s papers.
- ^11 ^Dr. Dunstaple also asks Fleury if he would fetch ‘half a dozen bottles of mustard’ during the novel (Farrell 2007, 161).
- ^12^ Lucy Hughes is Farrell’s interpretation of the ‘fallen woman’ referred to in Reverend Polehampton’s diary (Polehampton 1858, 108–109).
- ^13^ For a book about India, Indians themselves are woefully under represented in the novel, something Farrell later acknowledged with regret.
- ^14^ 1957 has a personal resonance for Farrell as well as a thematic one; the choice of year deliberately recalls the aftermath of the Suez Crisis, the widely accepted death-knell of Empire, and was also the year during which Farrell suffered his bout of polio.

## References

[CR1] Anonymous. 1854. *Leaves Turned Down or the Autobiography of an Indian Army Surgeon.* London: Richard Bentley of New Burlington Street

[CR2] Baly W, Gull WW (1854). Reports on Epidemic Cholera.

[CR3] Bhabha HK (2005). The Location of Culture.

[CR4] Boccardi M (2009). The Contemporary British Historical Novel: Representation, Nation, Empire.

[CR5] Bryden, James L. 1869. *Epidemic Cholera in the Bengal Presidency: A Report on the Cholera of 1866–68.* Calcutta.

[CR6] Byatt AS (2000). On Histories and Stories: Selected Essays.

[CR7] Carson, Ronald A. 2007. “Engaged Humanities: Moral Work in the Precincts of Medicine.” *Perspectives in Biology and Medicine* 50 (3). 10.1353/pbm.2007.0025 17660628

[CR8] Crane, Ralph and Jennifer Levitt. 1997. *Troubled Pleasures: The Fiction of J.G Farrell* Dublin: Four Courts Press.

[CR9] Collingham EM (2001). *Imperial Bodies*.

[CR10] Dean, Malcolm. 1973. “An Insight Job.” *The Guardian*, 1st September.

[CR11] ------. 1993. “A Personal Memoir.” In *The Hill Station* by J.G. Farrell. London: Phoenix.

[CR12] Eco U (1979). The Role of the Reader.

[CR13] Farrell JG (1993). The Hill Station.

[CR14] ------. 2007. *The Siege of Krishnapur.* London: Phoenix.

[CR15] ------. C. 1960–1979. Personal papers and manuscripts. Held by Trinity College Library, Dublin. Catalogue Reference 9138–9160.

[CR16] Forrest GW (1904). A History of the Indian Mutiny – Reviewed and Illustrated from Original Documents.

[CR17] Germon, MRS.R. C. 1870. *A Lady’s Diary During the Siege of Lucknow.* London: Waterlow & Sons Publishing

[CR18] Ghosh D (2006). Sex and the Family in Colonial India: The Making of Empire.

[CR19] Goonetilleke, D. C. R. A. 2003. “J.G Farrell’s Indian Works: His Majesty’s Subjects?” *Modern Asian Studies*, 37 (2).

[CR20] Greacen L (2000). J.G Farrell: The Making of a Writer.

[CR21] Hanen MP, Osler MJ, Weyant RG (1980). Science, Pseudo-science, and Society.

[CR22] Harrison M (2002). Climates & Constitutions: Health, Race, Environment & British Imperialism in India 1600–1850.

[CR23] Hartveit L (1993). The Imprint of Recorded Events in the Narrative Form of J.G Farrell’s The Siege of Krishnapur. English Studies.

[CR24] Hutcheon L (2002). The Politics of Postmodernism.

[CR25] James, Lawrence. 2007. *Raj: The Making and Unmaking of British Indi*a. London: Abacus.

[CR26] Jayapalan N (2005). History Of Education In India.

[CR27] Johnson, Alan. 2011. “Ghosts of Irish Famine in J. G. Farrell's *The Siege of Krishnapur*.” *The Journal of Commonwealth Literature* 46.

[CR28] Klein, Ira. 1980. “Cholera: Theory and Treatment in Nineteenth Century India.” *Journal of Indian History* 58.

[CR29] Magendie F (1843). An Elementary Treatise on Human Physiology.

[CR30] McLeod J (2007). J.G Farrell.

[CR31] Moore-Gilbert B (1994). Cultural Closure: The Arts in the 70s.

[CR32] Morey P (2000). Fictions of India: Narrative and Power.

[CR33] Nairn T (1979). The Break up of Britain: Crisis and NeoNationalism 1965–75.

[CR34] Paris, Alderson, Babington, Tweedie and Bagshaw-Ward. 1855. “General Board of Health Report On the Different Methods of Treatment Pursued in Epidemic Cholera in the Provinces Throughout England and Scotland in 1854”. London: George E. Eyre & William Spottiswoode.

[CR35] Pearse JS, Marston JA (1854). Statistics of the cases of the Cholera Epidemic 1853.

[CR36] Polehampton, Rev. H. S. 1858. *A Memoir, Letters & Diary of the Rev. Henry S. Polehampton, M.A.* London: Richard Bentley Publishing.

[CR37] Ross, J. T. C. C. 1850–1870. Memoranda, proposals, plans, inspection reports, instructions, etc., relating to military sanitation and the medical service in India. Wellcome Collection, London. Reference number: MS7858.

[CR38] Said E (2003). *Orientalism*.

[CR39] Singh Francis B. 1979. “Progress and History in J. G. Farrell’s *The Siege of Krishnapur.” Chandrabhaga* 2.

[CR40] Spivak G (1989). Selected Subaltern Studies.

[CR41] Spurling J (1993). “As Does the Bishop.” In *The Hill Station*.

[CR42] Strongman, Luke. 2002. “The Booker Prize & The Legacy of Empire.” *Cross Cultures* 54.

[CR43] Thornhill M (1884). The Indian Mutiny.

